# Characterization of Swine Influenza A(H1N2) Variant, Alberta, Canada, 2020

**DOI:** 10.3201/eid2712.210298

**Published:** 2021-12

**Authors:** Jamil N. Kanji, Kanti Pabbaraju, Matthew Croxen, Susan Detmer, Nathalie Bastien, Yan Li, Anna Majer, Hussein Keshwani, Nathan Zelyas, Ifeoma Achebe, Corinne Jones, Maureen Rutz, Angela Jacobs, Keith Lehman, Deena Hinshaw, Graham Tipples

**Affiliations:** Alberta Precision Laboratories, Edmonton, Alberta, Canada (J.N. Kanji, K. Pabbaraju, M. Croxen, N. Zelyas, G. Tipples);; University of Alberta, Edmonton (J.N. Kanji, M. Croxen, N. Zelyas, I. Achebe, D. Hinshaw, G. Tipples);; University of Saskatchewan, Regina, Saskatchewan, Canada (S. Detmer);; Public Health Agency of Canada, Winnipeg, Manitoba, Canada (N. Bastien, Y. Li, A. Majer);; Alberta Agriculture and Forestry, Edmonton (H. Keshwani, K. Lehman);; Alberta Health Services, Red Deer, Alberta, Canada (I. Achebe, C. Jones, M. Rutz);; Alberta Health Services, Edmonton (A. Jacobs);; University of Calgary, Calgary, Alberta, Canada (D. Hinshaw)

**Keywords:** influenza A, swine, variants, H1N2, pandemic potential, influenza, swine influenza, H1N2, zoonoses, viral zoonoses, zoonotic infections, respiratory infections, Canada, Alberta

## Abstract

Influenza strains circulating among swine populations can cause outbreaks in humans. In October 2020, we detected a variant influenza A subtype H1N2 of swine origin in a person in Alberta, Canada. We initiated a public health, veterinary, and laboratory investigation to identify the source of the infection and determine whether it had spread. We identified the probable source as a local pig farm where a household contact of the index patient worked. Phylogenetic analysis revealed that the isolate closely resembled strains found at that farm in 2017. Retrospective and prospective surveillance using molecular testing did not identify any secondary cases among 1,532 persons tested in the surrounding area. Quick collaboration between human and veterinary public health practitioners in this case enabled a rapid response to a potential outbreak.

Rapid detection and reporting of novel influenza A virus (IAV) strains are critical to prompt evaluation of a pandemic threat (*1*). For example, in 2009, health officials in Mexico reported a variant influenza A(H1N1) virus of swine origin; that variant quickly caused a pandemic (*2*). Although uncommon, transmission of swine variant IAV strains from pigs to humans has been documented on several occasions (*3*). Pig farming is structured such that the animals move to different types farms as they grow. Usually, piglets are born in farrowing farms, transferred to nurseries upon weaning, and then sent to finisher barns at 10–12 weeks of age. These farms usually span >2 geographically separate sites. Collectively, the combination of farrowing, nursery, and finisher farms form a chain where later farms are referred to as downstream from prior ones in the chain.

In 2005, updates to the International Health Regulations instituted mandatory reporting of pathogens such as novel influenza variants in all member states of the World Health Organization (*2*). Since then, 29 cases of swine influenza A(H1N2) variant H1N2v) strains have been reported to the World Health Organization, including 25 cases in the United States during 2011–2018, 1 case in Brazil in 2015, and 2 cases in Brazil in 2020 (*4*–*7*). In addition, 2 cases of H1N2v infection were detected in Canada: 1 in Alberta in October 2020 and 1 in Manitoba in April 2021 (*8*). Since the 2010–11 influenza season, the US Centers for Disease Control and Prevention has reported >465 cases of swine IAV variants, including H1N1v, H1N2v, and H3N2v, in humans (*9*). We report the detection and genetic characterization of an H1N2v IAV isolated from a patient in Alberta. We also describe the public health response and relevant investigations regarding the case.

## Methods

### Case Description

During the second week of October 2020, a child <18 years of age was brought to a local emergency department with a 4-day history of cough, fever, pharyngitis, and rhinorrhea. At admission, the patient was afebrile, had mild tachycardia and tachypnea, and had an oxygen saturation of 93% on ambient air. We did not find signs of respiratory distress and discharged the patient after collecting specimens for respiratory virus testing. The patient was born in Canada and up-to-date with all routine immunizations and influenza vaccinations until 2016.

Consent was provided for the patient and the patient’s household members to be included in this report provided that no identifiable information was published. Presentation of the data contained in this report has been approved by the Human Research Ethics Board at the University of Alberta (Edmonton, Alberta, Canada; study no. Pro00105933).

### Diagnostic Evaluation

The patient’s nasopharyngeal swab sample tested negative for severe acute respiratory syndrome coronavirus 2 (SARS-CoV-2) by real-time reverse transcription PCR (rRT-PCR) (*10*). Multiplex molecular respiratory virus testing (NxTAG Respiratory Pathogen Panel; Luminex Corporation, https://www.luminexcorp.com) detected the presence of IAV; however, the virus could not be subtyped. A second rRT-PCR confirmed the presence of IAV but not influenza A(H1N1)pdm09 virus (*11*,*12*). Partial Sanger sequencing of the hemagglutinin (HA) and neuraminidase (NA) genes using universal primers (*13*) yielded sequences that closely resembled isolates of swine H1N2 IAVs available in GenBank.

### Epidemiologic Inquiry

The patient had no history of travel, contact with persons from outside the county, or contact with persons who had respiratory illness. One of the patient’s household contacts worked with animals at a local pig farm. No household contacts reported symptoms of influenza-like illness or coronavirus disease (COVID-19) before the onset of illness in the index patient. However, 1 household contact reported influenza-like illness symptoms ≈2 days after symptom onset in the index patient. All household contacts remained at home for 10 days after symptom onset in the household contact. Both symptomatic persons recovered. Two asymptomatic household members, including one who worked at the farm of interest, consented to serologic testing by hemagglutination inhibition (HI) assay; samples were collected 35 days after identification of the index patient. The index patient and symptomatic household member declined serologic testing.

### Public Health Response

Upon confirmation of swine IAV, which occurred 3 weeks after collection of the original nasopharyngeal swab sample, provincial public health teams, in collaboration with the Alberta Precision Laboratories (Edmonton, Alberta, Canada), undertook heightened influenza surveillance measures in the geographic zone of Alberta where the case was identified. All respiratory specimens collected for community and hospital-based SARS-CoV-2 testing in that region during October 5–November 4, 2020, were retrieved from storage and tested for IAV (*12*). We conducted prospective testing for IAV on all respiratory specimens submitted for SARS-CoV-2 testing from that area during November 4–10, 2020. Patients being tested for SARS-CoV-2 were informed they would also be tested for IAV.

Upon notification of the case, the Office of the Chief Provincial Veterinarian (Edmonton, Alberta, Canada) began a veterinary investigation in collaboration with industry veterinarians and university partners to explore potential links to local pig herds. Staff of the Chief Provincial Veterinarian investigated the health, history and biosecurity practices of the farm where the household contact worked. Past samples collected from local pig herds showed IAVs of multiple subtypes in the farms supplying piglets to the herd of interest. In December 2017, a closely related H1N2 virus had been isolated from the nursery supplying the farm. In October 2019, a virus from the influenza A(H1N1)pdm09 clade was isolated from the nursery; this strain was most recently detected at the nursery in February 2020.

### Characterization of H1N2v Strain

We forwarded the patient’s sample to the National Microbiology Laboratory (Winnipeg, Manitoba, Canada) for isolation and further characterization. The virus was cultured on Madin-Darby canine kidney cells in 1 passage using standard techniques (*14*).

We conducted HI assays with 0.5% vol/vol turkey red blood cells and 4 HA units of A/Alberta/01/2020 (H1N2)v. We treated each serum with receptor-destroying enzyme (Denka Seiken, https://www.denka.co.jp) at a 1:4 dilution for 18 hrs at 37°C and 45 min at 56°C, then performed adsorption with packed turkey red blood cells (*15*). We defined the HI titer as the highest dilution of the serum capable of inhibiting hemagglutination.

We determined phenotypic susceptibility for oseltamivir and zanamivir by using a chemiluminescent NA inhibition assay (NA-Star Influenza Neuraminidase Inhibitor Resistance Detection Kit; Thermo Fisher Scientific, https://www.thermofisher.com) at the National Microbiology Laboratory. The assay used viruses standardized to equivalent NA enzyme activity and incubated with 0.0316–1,000 nmol of oseltamivir or zanamivir. We calculated the 50% inhibitory concentration (IC_50_) by plotting the percentage inhibition of NA activity against the inhibitor concentration, using PRISM version 4 (GraphPad Software, https://www.graphpad.com) for curve fitting.

We conducted whole-genome sequencing of the H1N2v isolate on the MinION (Oxford Nanopore Technologies, https://nanoporetech.com) and MiSeq (Illumina, https://www.illumina.com) platforms. We generated sequence data and prepared and sequenced libraries using the DNA Library Prep Kit and iSeq 100 (Illumina; Appendix). We conducted phylogenetic characterization of the H1N2v isolate by comparing human H1N2v and swine H1N2 HA (segment 4) sequences available on GenBank. We also aligned sequences and visualized the phylogenetic trees (Appendix).

### Sampling

We used the rope technique to collect samples from several farms, including the farm of interest (*16*). We also collected 56 deep nasal swab and 11 pen-based oral fluid samples from farms downstream of the farm of interest, as well as 12 nasal swab and 6 oral fluid samples from the farm of interest. We placed individual nasal swab samples in 1.5 mL of Dulbecco’s modified Eagle medium (Thermo Fisher Scientific) and vortexed them before extracting 500 µL pooled samples from 3–4 swabs for PCR (*17*). We subtyped the RNA from the strongest positive pooled nasal swab or oral fluid sample from each farm. Samples were analyzed at the University of Saskatchewan (Regina, Saskatchewan, Canada) by using the VetMAX-Gold Swine Influenza Virus Detection rRT-PCR kit and the VetMAX-Gold Swine Influenza Virus Subtyping rRT-PCR kit (Thermo Fisher Scientific) (*18*).

## Results

BLAST (https://blast.ncbi.nlm.nih.gov/Blast.cgi) analysis of historical swine H1N2 and H1N2 isolates from western Canada yielded a close match to a virus strain found on only a few farms in central Alberta, including a farm in the pig supply chain of the herd of interest. Phylogenetic analysis showed that the human A/Alberta/01/2020 H1N2v isolate belonged to the evolutionary branch found at the nursery that supplied the farm where the household contact worked (A/swine/Alberta/SD0237/2017 and A/swine/Alberta/SD0267/2017); the most similar sequence was collected from the source farm in 2017 (Figure; Appendix Table). The A/Alberta/01/2020 H1N2v sequence shared high genetic similarity (98%–>99%) with 8 genes from multiple swine H1N2v and H3N2 strains from western Canada and the United States (Table). The H1 gene sequence most resembled sequences found in the H1α-3a subclade.

**Figure:**
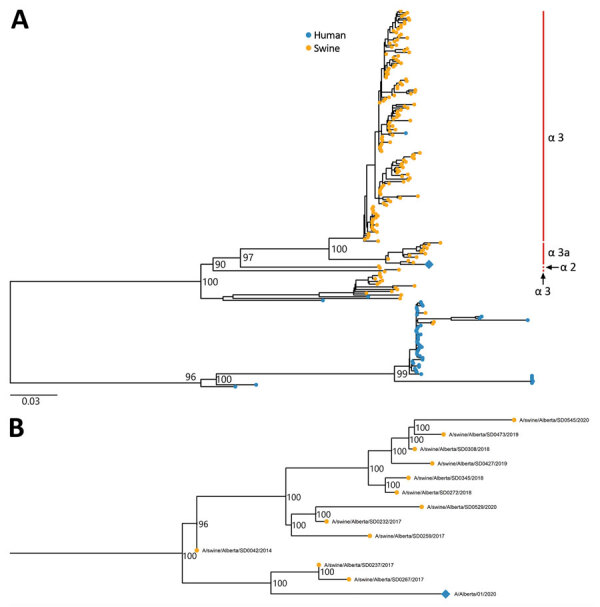
Phylogenetic trees of A/Alberta/01/2020 H1N2v and related strains, United States and Canada, 2016–2020. A) The H1α-3a subclade. B) The H1α-3a subclade with ≈2 years of changes (2017–2018) of A/swine/Alberta/SD0237/2017 and A/swine/Alberta/SD0267/2017. The trees were built with IQ-TREE version 2.0.3 (http://www.iqtree.org) on the basis of hemagglutinin sequences. Numbers at nodes indicate bootstrap values based on 1,000 replicates. Red bars identifies clades; all clades presented have % age bootstrap values >70. Diamond indicates A/Alberta/01/2020 (H1N2)v. Scale bar indicates number of nucleotide substitutions per site.

**Table:** Genetic comparison of A/Alberta/01/2020 (H1N2)v and other influenza strains in swine and humans, United States and Canada, 2016–2020*

GISAID ID	Segment	Gene	GenBank accession no.	% Identity	Host	Genotype	Country	Strain	Clade
EPI1815176	1	PB2	MK475180	99.3	Human	H1N1	USA	A/Connecticut/37/2018	npdm
			MK475443	99.3	Human	H1N1	USA	A/Montana/39/2018	npdm
			MK623898	99.3	Human	H1N1	USA	A/Wisconsin/517/2018	npdm
EPI1056722				91.4	Human	H1N2v	USA	A/Ohio/24/2017†	H1α-3 variant
EPI1815177	2	PB1	MK631128	99.3	Human	H1N1	USA	A/Iowa/01/2019	npdm
EPI1056723				91.3	Human	H1N2v	USA	A/Ohio/24/2017†	H1α-3 variant
EPI1815175	3	PA	MK462359	98.7	Swine	H3N2	Canada	A/swine/Saskatchewan/ SD0247/2017	Swine H3
			MK462496	98.7	Swine	H3N2	Canada	A/swine/Saskatchewan/ SD0258/2017	Swine H3
EPI1056721				96.4	Human	H1N2v	USA	A/Ohio/24/2017†	H1α-3 variant
EPI1815179	4	HA	MK462499	97.9	Swine	H1N2	Canada	A/swine/Alberta/SD0237/2017	H1α-3a
EPI1056725				91.7	Human	H1N2v	USA	A/Ohio/24/2017†	H1α-3 variant
EPI1815172	5	NP	MK445889	99.0	Human	H1N1	USA	A/North Dakota/42/2018	npdm
EPI1056718				94.9	Human	H1N2v	USA	A/Ohio/24/2017†	H1α-3 variant
EPI1815178	6	NA	MK462493	98.2	Swine	H3N2	Canada	A/swine/Saskatchewan/ SD0258/2017	Swine H3
EPI1056724				95.4	Human	H1N2v	USA	A/Ohio/24/2017†	H1α-3 variant
EPI1815174	7	M	CY246708	99.5	Swine	H1N2	Canada	A/swine/Saskatchewan/ SD0204/2016H1N2	H1α-3
			CY246716	99.5	Swine	H1N2	Canada	A/swine/Manitoba/SD0203/ 2016H1N2	H1α-3
			MK462355	99.5	Swine	H3N2	Canada	A/swine/Saskatchewan/ SD0247/2017	Swine H3
EPI1056720				96.6	Human	H1N2v	Canada	A/Ohio/24/2017†	H1α-3 variant
EPI1815173	8	NS	MK462463	99.2	Swine	H1N2	Canada	A/swine/Alberta/SD0267/2017	H1α-3a
EPI1056719				94.1	Human	H1N2v	USA	A/Ohio/24/2017†	H1α-3 variant

*GISAID, https://www.gisaid.org. HA, hemagglutinin; ID, identification; M, matrix; NA, neuraminidase; NP, nucleoprotein; npdm, influenza A(H1N1)pdm09-like virus; NS, nonstructural protein; PA, polymerase acidic protein; PB1, polymerase base protein 1; PB2, polymerase basic protein 2. †A/Ohio/24/2017 data was deposited into GISAID by the Centers for Disease Control (Atlanta, Georgia, USA) and first identified in the Ohio Department of Health Laboratories (Reynoldsburg, Ohio, USA).

Two members of patient’s household had HI titers of 20. Seasonal vaccine serum for A/Hawaii/70/2019 H1N1 elicited no HI titer against A/Alberta/01/2020 H1N2v. We found that A/Alberta/01/2020/H1N2v was susceptible to oseltamivir (IC_50_ 0.41 nmol) and zanamivir (IC_50_ 2.16 nmol). The control strain, A/Brisbane/10/07 H3N2, was sensitive to oseltamivir (IC_50_ 0.61 nmol) and zanamivir (IC_50_ 3.19 nmol).

### Surveillance for Additional H1N2v Cases

This case occurred during the response to the COVID-19 pandemic, when there was no circulating seasonal influenza in the province (*19*). We did not identify additional cases of IAV from retrospective testing of 1,276 archived respiratory specimens nor prospective testing of 256 specimens submitted for respiratory virus testing.

### IAV Testing

In total, 3/13 pooled nasal swab and 2/6 oral fluid samples from the farm of interest tested positive for IAV. Cycle threshold (C_t_) values were 34.7–36.6; we considered values <38 to be positive. We found that all positive pooled nasal swab and oral fluid samples from the 2 sites of interest with the lowest C_t_ value (34.7) had the H1 gene; however, we could not identify the NA type. The sample with the second lowest C_t_ value (35.4) from the same downstream site was subtyped as N2, as was the only positive (C_t_ 36.0) sample from the other downstream farm tested.

At the nursery, all 3 pooled nasal swab and all 6 oral fluid samples tested positive for IAV (C_t_ 29.8–34.7). We identified H1, H3, N1, and N2 genes in the pooled nasal swab sample with the lowest C_t_ value (29.8) from the nursery.

## Discussion

We report pig-to-human transmission of H1N2v virus in Alberta, Canada. The source of H1N2v infection in the index patient is unclear, but one of the patient’s household contacts worked at a pig farm where a similar H1N2 virus was found in 2017.

IAV is transmitted through close contact and contaminated objects (*5*). Transmission of IAV from swine to humans is usually the result of close contact or self-inoculation from contaminated farm surfaces (*3*,*20*,*21*). In Canada, pig farms must adhere to strict farm biosecurity measures, such as policies requiring workers to shower before leaving, use of facility-specific uniforms and boots, and designation of clean and dirty zones (*22*–*24*). Thus, the likelihood of the index patient acquiring infection through fomite-related transmission is very low. Because the index patient never visited the farm of interest, the virus was probably transmitted through respiratory droplets from the household contact who worked at the farm. Humans have poor seroconversion to H1N2 viruses (*20*), so it is possible that the household contact had partial protection from previous exposure, despite having an HI titer of 20. Partial protection might also explain the limited forward transmission observed in this study, especially because H1N2v virus is associated with mild illness and limited transmission (*5*).

Although not an exact predictor of protection, HI titers of 40 are considered the minimum protective level for humans (*25*). Therefore, titers of 20 against the isolated strain would not indicate a recent stimulation of the antibodies or a protective cross-reaction. However, a very mild or asymptomatic infection might not result in a high titer immune response (*26*). Because testing with A/Hawaii/70/2019 (H1N1) antiserum elicited no titer, seasonal vaccination would probably not provide protection against A/Alberta/01/2020/H1N2v.

Extensive surveillance testing in the geographic area of the index patient did not detect additional cases, indicating minimal spread. After detection of a variant influenza, active case-finding is critical because of the potential pandemic threat posed by emerging strains. A comparative evaluation of H1N1v and H1N2v viruses isolated during 2011–2016 found that many swine H1 strains capable of infecting humans possess adaptations for efficient replication and enhanced transmission through respiratory droplets (*27*). Furthermore, the H1 antigens in most isolates were distinct from those of vaccine strains; thus, routine influenza vaccine is unlikely to provide adequate protection against variant strains. Even 1 zoonotic event could enable adaptations for human infection and transmission (*28*).

We found low levels of circulating virus among pigs at the farm where the index patient’s household member worked and those downstream from it, possibly because the 2 sites were finisher barns where medium-sized grower pigs are raised until they reach market weight. Sows who have antibodies to IAV from vaccination or natural exposure can pass maternally derived antibodies to their offspring through colostrum. The amount of maternally derived antibodies detected in newborn piglets varies within litters, not only because antibodies vary among sows, but also because the firstborn piglets consume the most colostrum. In total, 60%–100% of nursing piglets born to a vaccinated sow have HI titers >120 at 5 days old. On average, 75% of the nursing piglets of vaccinated sows have a strong titer (>120) that is homologous between the vaccine and circulating strains (i.e., autogenous vaccines) (S. Detmer, University of Saskatchewan, pers. comm., 2021 Jun 5). As such, there is limited detection of virus in farrowing barns. In contrast, IAVs are often detected in and isolated from nursing piglets 14–24 days of age on farms that do not vaccinate sows, further demonstrating that maternally derived antibodies from vaccinated sows limit infection in nursing piglets until these antibodies wane at 6–8 weeks of age (*29*). By the time pigs reach the finisher barns, they often have experienced infection in the nursery, farrowing barn, or both and have acquired their own immune response to endemic strains of IAV. In finisher barns, acquired immunity among pigs causes many IAV infections to be subclinical and undetected (*30*).

In 2016, analysis of whole influenza genomes isolated from pigs in Canada and the United States demonstrated the splitting of the H1α clade into 3 distinct subclades: H1α-1, H1α-2, and H1α-3 (*31*). We found that A/Alberta/01/2020/H1N2v most closely resembles strains belonging to the H1α-3 virus clade; however, the variant strain does not contain the amino acid deletions at sites 129 and 130 in the HA gene. Therefore, A/Alberta/01/2020/H1N2v probably belongs to the H1α-3a virus clade. Further study is required to assess if the H1α-3a virus cluster is antigenically in addition to genetically distinct from the H1α-3 virus cluster.

A zoonotic H1α-3 H1N2v virus strain (A/Ohio/24/2017/H1N2) was isolated from a human in the United States in 2017 (*32*). The A/Ohio/24/2017/H1N2 and A/Alberta/01/2020 strains share high nucleotide identity (91.3%–96.6%) within 8 major genes; however, this proportion is lower than that of other H1N2 strains (Table). As of July 2021, 9 distinct H1α-3a viruses have been detected in the United States. These 9 viruses were probably associated with animal movements from Alberta to the US states of Iowa and South Dakota. These animal movements stopped in 2018 and there is no evidence of continued transmission or geographic spread of these strains in the United States (*31*).

A major limitation of this study is the 3-week delay in confirming the patient’s H1N2v infection. Therefore, active surveillance testing of persons and pigs in the patient’s geographic region was delayed, possibly diminishing our ability to detect additional active cases. However, we mitigated this limitation in human sampling by testing banked samples collected for SARS-CoV-2 testing beginning ≈1 week before the collection of the patient’s nasopharyngeal swab sample. The delay might have contributed to the higher C_t_ values for IAV testing of samples from pigs at the farm of interest, because the active phase of infection probably occurred several weeks earlier in most pigs. The higher C_t_ values of >30 also decreased our ability to amplify certain gene segments for strain identification.

As of September 2021, no further H1N2v virus cases have been identified in humans in that area of Alberta. This case occurred when IAV incidence among humans was uncharacteristically low, probably because of nonpharmaceutical interventions implemented in response to the COVID-19 pandemic (*33*). These interventions simplified IAV screening. Our results highlight the importance of expanding collaborations between the human and veterinary sectors to enable timely identification, reporting, and investigation of emerging zoonotic pathogens of pandemic potential.

Appendix Additional information on characterization of swine influenza A(H1N2) variant, Alberta, Canada, 2020.
