# Supplementary material for: Characterization of Swine Influenza A(H1N2) Variant, Alberta, Canada, 2020
Source: Emerg Infect Dis. 2021 Dec;27(12):3045–51. doi: 10.3201/eid2712.210298 (PMC8632177; doi:10.3201/eid2712.210298)
Supplement: Appendix — Additional information on characterization of swine influenza A(H1N2) variant, Alberta, Canada, 2020. [file 21-0298-Techapp-s1.pdf]

Article DOI: https://doi.org/10.3201/eid2712.210298

Characterization of Swine Influenza A(H1N2) Variant, Alberta, Canada, 2020

Appendix

Appendix Methods

Whole-Genome Sequencing

To generate MinION (Oxford Nanopore Technologies, https://nanoporetech.com) sequence data, reverse transcription PCR was performed using SuperScript IV First-Strand Synthesis System (Thermo Fisher Scientific, https://www.thermofisher.com) as previously described (P.C. Resende, unpub. data, https://www.biorxiv.org/content/10.1101/2020.04.30.069039v1). Each influenza genome segment was amplified by using universal influenza primers (*1*) and Q5 High-Fidelity DNA Polymerase (New England Biolabs, https://www.neb.com) with the following thermocycler conditions: 98°C for 30 s, 40 cycles of 98°C for 17 s, 55°C for 30 s, 72°C for 2 min, and final extension at 72°C for 4 min. PCR products were purified by using Agencourt AMPure XP beads (Beckman Coulter, Inc., https://www.beckmancoulter.com) as per manufacturer's recommendations and DNA concentration was measured by using the Qubit 4 Fluorometer (Thermo Fisher Scientific) with the Qubit dsDNA Broad Range Assay Kit (Thermo Fisher Scientific). Sequencing libraries were prepared using Ligation Sequencing Kit 1D (SQK-LSK109) and the Native Barcoding Expansion 1–24 (Oxford Nanopore Technologies) as per manufacturer's recommendations and previously described protocol (P.C. Resende, unpub. data, https://www.biorxiv.org/content/10.1101/2020.04.30.069039v1). In brief, 60 ng of each amplicon were used to prepare sequencing libraries. Sequencing was performed by using the FLO-MIN106D flow cell R9.4.1 for 24 hours on the GridION (Oxford Nanopore Technologies). MinKNOW version 20.06.17 (Oxford Nanopore Technologies) was used to generate high-accuracy base calling. Reads with a quality score of >7 and dual barcodes were selected for further analysis.

Nanopore data analysis was performed by using CLCGenomics Workbench version 20.0.4 (QIAGEN, https://www.qiagen.com). Reads were filtered by length where segments within ±200 bp of the specified viral genome segment length were retained and barcodes were trimmed from the data. Trimmed reads were mapped against H1N2 virus reference sequence (GenBank accession no. MK462499.1) using the Map Long Reads to Reference tool (default settings). Consensus was called using the Extract Consensus Sequence tool (default settings) with a minimum coverage of 50 reads.

The MinION-generated consensus for the influenza PA gene segment was further polished using Illumina (https://www.illumina.com) data. In brief, dsDNA was synthesized using Maxima H Minus Double-Stranded cDNA Synthesis Kit (Thermo Fisher Scientific), per manufacturer's recommendation using random hexamers. Libraries were prepared using the Nextera DNA Flex Library Prep (Illumina) following manufacturer's recommendations. The pooled library was sequenced using the iSeq-100 platform (Illumina) to generate 2 × 150 bp paired-end reads. Reads were filtered by quality and adapters trimmed. The resulting data were used to polish the nanopore consensus using the Polish with Reads tool (default settings).

Phylogenetic Characterization

In total, 202 H1N2 and H1N2v hemagglutinin (segment 4) complimentary DNA sequences from 47 humans in the United States and Canada and 155 swine in Canada were extracted from the NCBI Influenza Virus Database (https://www.ncbi.nlm.nih.gov; cited 2021 Jan 14) and were aligned with A/Alberta/01/2020 H1N2v and 6 additional previously unpublished Alberta swine hemagglutinin sequences using MAFFT version 7.475 (*2*). This alignment was used as the input to generate a maximum-likelihood tree using IQ-TREE version 2.0.3 (*3*) with 1,000 bootstrap replicates (*4*) and 1,000 SH-like approximate likelihood ratio tests (*5*) and the TVM+F+I+G4 model on ModelFinder (*6*). The tree was visualized with ggtree (*7*). The 8 segments from A/Alberta/01/2020 H1N2v were aligned with sequences from the Influenza Virus Database using blastn version 2.10.1+ (*8*) to identify the alignment with the highest bit score. Alberta/01/2020 H1N2v was uploaded to GISAID (https://www.gisaid.org) under accession nos. EPI1815172–EPI1815179.

References

1. Hoffmann E, Stech J, Guan Y, Webster RG, Perez DR. Universal primer set for the full-length amplification of all influenza A viruses. Arch Virol. 2001;146:2275–89. PubMed https://doi.org/10.1007/s007050170002

2. Katoh K, Standley DM. MAFFT multiple sequence alignment software version 7: improvements in performance and usability. Mol Biol Evol. 2013;30:772–80. PubMed https://doi.org/10.1093/molbev/mst010

3. Minh BQ, Schmidt HA, Chernomor O, Schrempf D, Woodhams MD, von Haeseler A, et al. IQ-TREE 2: new models and efficient methods for phylogenetic inference in the genomic era. Mol Biol Evol. 2020;37:1530–4. PubMed https://doi.org/10.1093/molbev/msaa015

4. Hoang DT, Chernomor O, von Haeseler A, Minh BQ, Vinh LS. UFBoot2: improving the ultrafast bootstrap approximation. Mol Biol Evol. 2018;35:518–22. PubMed https://doi.org/10.1093/molbev/msx281

5. Guindon S, Dufayard JF, Lefort V, Anisimova M, Hordijk W, Gascuel O. New algorithms and methods to estimate maximum-likelihood phylogenies: assessing the performance of PhyML 3.0. Syst Biol. 2010;59:307–21. PubMed https://doi.org/10.1093/sysbio/syq010

6. Kalyaanamoorthy S, Minh BQ, Wong TKF, von Haeseler A, Jermiin LS. ModelFinder: fast model selection for accurate phylogenetic estimates. Nat Methods. 2017;14:587–9. PubMed https://doi.org/10.1038/nmeth.4285

7. Yu G, Lam TT, Zhu H, Guan Y. Two methods for mapping and visualizing associated data on phylogeny using ggtree. Mol Biol Evol. 2018;35:3041–3. PubMed https://doi.org/10.1093/molbev/msy194

8. Altschul SF, Gish W, Miller W, Myers EW, Lipman DJ. Basic local alignment search tool. J Mol Biol. 1990;215:403–10. PubMed https://doi.org/10.1016/S0022-2836(05)80360-2

Appendix Table. Influenza A strains used in phylogenetic analysis of A/Alberta/01/2020 (H1N2)v, Canada, 2020*

NCBI accession no.	Host	Genotype	Strain	Clade
FJ231789	Human	H1N2	A/Baden-Wuerttemberg/129/2003	δ-like HA
FJ231788	Human	H1N2	A/Baden-Wuerttemberg/20/2003	δ-like HA
MK239097	Human	H1N2v	A/California/58/2018	δ2 variant
MK239094	Human	H1N2v	A/Colorado/16/2017	δ1 variant
EU097961	Human	H1N2	A/Denmark/12/2003	δ-like HA
EU097962	Human	H1N2	A/Denmark/50/2003	δ-like HA
EU097960	Human	H1N2	A/Denmark/56/2003	δ-like HA
EU097959	Human	H1N2	A/Denmark/86/2003	δ-like HA
JQ340003	Human	H1N2	A/Eastern India/N-1289/2009	npdm
CY122316	Human	H1N2	A/Memphis/8/2003	δ-like HA
CY016324	Human	H1N2	A/Michigan/2/2003	δ-like HA
CY016326	Human	H1N2	A/Michigan/3/2003	δ-like HA
MK239117	Human	H1N2v	A/Michigan/382/2018	δ2 variant
MK239081	Human	H1N2v	A/Michigan/383/2018	δ2 variant
MK239107	Human	H1N2v	A/Michigan/384/2018	δ2 variant
KJ620415	Human	H1N2	A/Minnesota/14/2012	δ-like HA
JQ290156	Human	H1N2	A/Minnesota/19/2011	δ-like HA
CY045844	Human	H1N2	A/NWS-FNWS/1934-Rockefeller Institute/5/1957	Not available
CY002352	Human	H1N2	A/New York/209/2003	δ-like HA
CY006867	Human	H1N2	A/New York/211/2003	δ-like HA
CY003368	Human	H1N2	A/New York/217/2002	δ-like HA
CY006107	Human	H1N2	A/New York/219/2003	δ-like HA
CY006747	Human	H1N2	A/New York/225/2003	δ-like HA
CY002992	Human	H1N2	A/New York/226/2003	δ-like HA
CY010076	Human	H1N2	A/New York/229/2003	δ-like HA
CY002632	Human	H1N2	A/New York/231/2003	δ-like HA
CY002656	Human	H1N2	A/New York/294/2003	δ-like HA
CY002152	Human	H1N2	A/New York/296/2003	δ-like HA
CY002664	Human	H1N2	A/New York/297/2003	δ-like HA
CY002360	Human	H1N2	A/New York/300/2003	δ-like HA
CY003761	Human	H1N2	A/New York/400/2003	δ-like HA
CY003769	Human	H1N2	A/New York/417/2002	δ-like HA
CY006387	Human	H1N2	A/New York/481/2003	δ-like HA
CY003672	Human	H1N2	A/New York/482/2003	δ-like HA
CY006395	Human	H1N2	A/New York/487/2003	δ-like HA
CY003696	Human	H1N2	A/New York/489/2003	δ-like HA
CY006403	Human	H1N2	A/New York/490/2003	δ-like HA
CY006411	Human	H1N2	A/New York/492/2003	δ-like HA
CY001680	Human	H1N2	A/New York/78/2002	δ-like HA
CY017115	Human	H1N2	A/New York/C1/2003	δ-like HA
MK239115	Human	H1N2v	A/Ohio/24/2018	δ2 variant
CY146817	Human	H1N2	A/PAL/unknown	Not available
EF101741	Human	H1N2	A/Philippines/344/2004	H1α-like HA
FJ231790	Human	H1N2	A/Rheinland-Pfalz/34/2003	δ-like HA
FJ231787	Human	H1N2	A/Sachsen/11.03.02/2002	δ-like HA
FJ231786	Human	H1N2	A/Sachsen/05.03.02/2002	δ-like HA
FJ231791	Human	H1N2	A/Sachsen/678/2003	δ-like HA
CY195359	Swine	H1N2	A/swine/Alberta/SD0042/2014	H1α-3a
CY246227	Swine	H1N2	A/swine/Alberta/SD0164/2016	H1α-3
MK462854	Swine	H1N2	A/swine/Alberta/SD0217/2017	npdm
MK462483	Swine	H1N2	A/swine/Alberta/SD0222/2017	H1α-3
MK462750	Swine	H1N2	A/swine/Alberta/SD0227/2017	H1α-3
MK462667	Swine	H1N2	A/swine/Alberta/SD0228/2017	H1α-3
MK462798	Swine	H1N2	A/swine/Alberta/SD0232/2017	H1α-3a
MK462499	Swine	H1N2	A/swine/Alberta/SD0237/2017	H1α-3a
MK462619	Swine	H1N2	A/swine/Alberta/SD0246/2017	H1α-3
MK462846	Swine	H1N2	A/swine/Alberta/SD0259/2017	H1α-3a
MK462459	Swine	H1N2	A/swine/Alberta/SD0267/2017	H1α-3a
MK462290	Swine	H1N2	A/swine/Alberta/SD0272/2018	H1α-3a
CY194381	Swine	H1N2	A/swine/Manitoba/D0104/2012	npdm
CY194237	Swine	H1N2	A/swine/Manitoba/D0170/2012	npdm
CY194277	Swine	H1N2	A/swine/Manitoba/D0171/2012	npdm
CY194309	Swine	H1N2	A/swine/Manitoba/D0173/2012	npdm
CY194597	Swine	H1N2	A/swine/Manitoba/D0226/2013	npdm
CY194765	Swine	H1N2	A/swine/Manitoba/D0265/2013	H1α-3
CY194781	Swine	H1N2	A/swine/Manitoba/D0267/2013	H1α-3
CY194789	Swine	H1N2	A/swine/Manitoba/D0268/2013	H1α-3
CY194829	Swine	H1N2	A/swine/Manitoba/D0272/2013	H1α-3

NCBI accession no.	Host	Genotype	Strain	Clade
CY194837	Swine	H1N2	A/swine/Manitoba/D0273/2013	H1α-3
CY194853	Swine	H1N2	A/swine/Manitoba/D0275/2013	H1α-3
CY194861	Swine	H1N2	A/swine/Manitoba/D0276/2013	H1α-3
CY194869	Swine	H1N2	A/swine/Manitoba/D0277/2013	H1α-3
CY194885	Swine	H1N2	A/swine/Manitoba/D0279/2013	H1α-3
CY194909	Swine	H1N2	A/swine/Manitoba/D0282/2013	H1α-3
CY194925	Swine	H1N2	A/swine/Manitoba/D0284/2013	H1α-3
CY194933	Swine	H1N2	A/swine/Manitoba/D0285/2013	H1α-3
CY194941	Swine	H1N2	A/swine/Manitoba/D0286/2013	H1α-3
CY194966	Swine	H1N2	A/swine/Manitoba/D0289/2013	H1α-3
CY194974	Swine	H1N2	A/swine/Manitoba/D0290/2013	H1α-3
CY194998	Swine	H1N2	A/swine/Manitoba/D0293/2013	H1α-3
CY195006	Swine	H1N2	A/swine/Manitoba/D0294/2013	H1α-3
CY195014	Swine	H1N2	A/swine/Manitoba/D0295/2013	H1α-3
CY195030	Swine	H1N2	A/swine/Manitoba/D0296/2013	H1α-3
CY195038	Swine	H1N2	A/swine/Manitoba/D0297/2013	H1α-3
CY195046	Swine	H1N2	A/swine/Manitoba/D0298/2013	H1α-3
CY195062	Swine	H1N2	A/swine/Manitoba/D0301/2013	H1α-3
CY195070	Swine	H1N2	A/swine/Manitoba/D0302/2013	H1α-3
CY195102	Swine	H1N2	A/swine/Manitoba/D0303/2014	H1α-3
CY195126	Swine	H1N2	A/swine/Manitoba/D0316/2014	H1α-3
CY195134	Swine	H1N2	A/swine/Manitoba/D0317/2014	H1α-3
CY195142	Swine	H1N2	A/swine/Manitoba/D0319/2014	H1α-3
CY195167	Swine	H1N2	A/swine/Manitoba/D0322/2014	H1α-3
CY195175	Swine	H1N2	A/swine/Manitoba/D0324/2014	H1α-3
CY195199	Swine	H1N2	A/swine/Manitoba/D0328/2014	H1α-3
CY195575	Swine	H1N2	A/swine/Manitoba/D0333/2014	H1α-3
CY195583	Swine	H1N2	A/swine/Manitoba/D0335/2014	H1α-3
CY195215	Swine	H1N2	A/swine/Manitoba/D0336/2014	H1α-3
CY195223	Swine	H1N2	A/swine/Manitoba/D0337/2014	H1α-3
CY195231	Swine	H1N2	A/swine/Manitoba/D0338/2014	npdm
CY195239	Swine	H1N2	A/swine/Manitoba/D0339/2014	H1α-3
CY195255	Swine	H1N2	A/swine/Manitoba/D0341/2014	H1α-3
CY195263	Swine	H1N2	A/swine/Manitoba/D0342/2014	H1α-2
CY195279	Swine	H1N2	A/swine/Manitoba/D0345/2014	H1α-3
CY195287	Swine	H1N2	A/swine/Manitoba/D0347/2014	H1α-3
CY195295	Swine	H1N2	A/swine/Manitoba/D0348/2014	H1α-3
CY195319	Swine	H1N2	A/swine/Manitoba/D0351/2014	H1α-3
CY195375	Swine	H1N2	A/swine/Manitoba/D0357/2014	H1α-3
CY195383	Swine	H1N2	A/swine/Manitoba/D0358/2014	H1α-3
CY195407	Swine	H1N2	A/swine/Manitoba/D0359/2014	H1α-3
CY195591	Swine	H1N2	A/swine/Manitoba/D0361/2014	H1α-3
CY195423	Swine	H1N2	A/swine/Manitoba/D0362/2014	H1α-3
CY194325	Swine	H1N2	A/swine/Manitoba/D0364/2012	npdm
CY194373	Swine	H1N2	A/swine/Manitoba/D0365/2012	npdm
CY195495	Swine	H1N2	A/swine/Manitoba/D0369/2015	H1α-3
CY195511	Swine	H1N2	A/swine/Manitoba/D0372/2015	H1α-3
CY195527	Swine	H1N2	A/swine/Manitoba/D0374/2015	H1α-3
CY195543	Swine	H1N2	A/swine/Manitoba/D0377/2015	H1α-3
CY195551	Swine	H1N2	A/swine/Manitoba/D0378/2015	H1α-3
CY245833	Swine	H1N2	A/swine/Manitoba/D0389/2015	H1α-3
CY245697	Swine	H1N2	A/swine/Manitoba/D0391/2015	H1α-3
CY245769	Swine	H1N2	A/swine/Manitoba/D0399/2015	H1α-3
CY245889	Swine	H1N2	A/swine/Manitoba/D0401/2015	H1α-3
CY245953	Swine	H1N2	A/swine/Manitoba/D0402/2015	H1α-3
CY245897	Swine	H1N2	A/swine/Manitoba/D0404/2015	H1α-3
CY245905	Swine	H1N2	A/swine/Manitoba/D0405/2015	H1α-3
CY245937	Swine	H1N2	A/swine/Manitoba/D0406/2015	H1α-3
CY245929	Swine	H1N2	A/swine/Manitoba/D0407/2015	H1α-3
CY246129	Swine	H1N2	A/swine/Manitoba/D0409/2016	H1α-3
CY246235	Swine	H1N2	A/swine/Manitoba/D0417/2016	H1α-3
CY246275	Swine	H1N2	A/swine/Manitoba/D0424/2016	H1α-3
CY246291	Swine	H1N2	A/swine/Manitoba/D0426/2016	H1α-3
CY245961	Swine	H1N2	A/swine/Manitoba/D0432/2015	H1α-3
CY246033	Swine	H1N2	A/swine/Manitoba/D0434/2016	H1α-3
CY246041	Swine	H1N2	A/swine/Manitoba/D0435/2016	H1α-3
CY246049	Swine	H1N2	A/swine/Manitoba/D0436/2016	H1α-3
CY246057	Swine	H1N2	A/swine/Manitoba/D0437/2016	H1α-3
CY246324	Swine	H1N2	A/swine/Manitoba/D0443/2016	H1α-3

NCBI accession no.	Host	Genotype	Strain	Clade
CY246340	Swine	H1N2	A/swine/Manitoba/D0445/2016	H1α-3
CY246348	Swine	H1N2	A/swine/Manitoba/D0446/2016	H1α-3
CY246356	Swine	H1N2	A/swine/Manitoba/D0447/2016	H1α-3
CY246380	Swine	H1N2	A/swine/Manitoba/D0448/2016	H1α-3
CY246428	Swine	H1N2	A/swine/Manitoba/D0450/2016	H1α-3
CY246436	Swine	H1N2	A/swine/Manitoba/D0451/2016	H1α-3
CY246500	Swine	H1N2	A/swine/Manitoba/D0456/2016	H1α-3
CY246508	Swine	H1N2	A/swine/Manitoba/D0457/2016	H1α-3
CY246580	Swine	H1N2	A/swine/Manitoba/D0462/2016	H1α-3
CY246588	Swine	H1N2	A/swine/Manitoba/D0463/2016	H1α-3
CY246628	Swine	H1N2	A/swine/Manitoba/D0468/2016	H1α-3
CY246636	Swine	H1N2	A/swine/Manitoba/D0469/2016	H1α-3
CY246731	Swine	H1N2	A/swine/Manitoba/D0477/2016	H1α-3
MK462734	Swine	H1N2	A/swine/Manitoba/D0479/2017	H1α-3
MK462902	Swine	H1N2	A/swine/Manitoba/D0483/2017	H1α-3
MK462742	Swine	H1N2	A/swine/Manitoba/D0484/2017	H1α-3
MK462346	Swine	H1N2	A/swine/Manitoba/D0487/2017	H1α-3
MK462571	Swine	H1N2	A/swine/Manitoba/D0489/2017	H1α-3
MK462651	Swine	H1N2	A/swine/Manitoba/D0494/2017	H1α-3
MK462603	Swine	H1N2	A/swine/Manitoba/D0495/2017	H1α-3
CY246203	Swine	H1N2	A/swine/Manitoba/D0501/2016	H1α-3
MK462950	Swine	H1N2	A/swine/Manitoba/D0511/2017	H1α-3
MK462635	Swine	H1N2	A/swine/Manitoba/D0519/2017	H1α-3
MK462595	Swine	H1N2	A/swine/Manitoba/D0523/2017	H1α-3
MK462379	Swine	H1N2	A/swine/Manitoba/D0526/2017	H1α-3
MK328701	Swine	H1N2	A/swine/Manitoba/DM_08/2016	H1α-3
KX264745	Swine	H1N2	A/swine/Manitoba/G2/2014	H1α-3
KX264747	Swine	H1N2	A/swine/Manitoba/G4/2014	H1α-3
KX264749	Swine	H1N2	A/swine/Manitoba/G6/2014	H1α-3
CY246001	Swine	H1N2	A/swine/Manitoba/SD0129/2015	H1α-3
CY246073	Swine	H1N2	A/swine/Manitoba/SD0133/2016	H1α-3
CY246105	Swine	H1N2	A/swine/Manitoba/SD0144/2016	H1α-3
CY246153	Swine	H1N2	A/swine/Manitoba/SD0148/2016	H1α-3
CY246145	Swine	H1N2	A/swine/Manitoba/SD0150/2016	H1α-3
CY245592	Swine	H1N2	A/swine/Manitoba/SD0157/2016	H1α-3
CY246187	Swine	H1N2	A/swine/Manitoba/SD0159/2016	H1α-3
CY245609	Swine	H1N2	A/swine/Manitoba/SD0165/2016	H1α-3
CY245617	Swine	H1N2	A/swine/Manitoba/SD0166/2016	H1α-3
CY246396	Swine	H1N2	A/swine/Manitoba/SD0170/2016	H1α-3
CY246388	Swine	H1N2	A/swine/Manitoba/SD0171/2016	H1α-3
CY246723	Swine	H1N2	A/swine/Manitoba/SD0202/2016	H1α-3
CY246715	Swine	H1N2	A/swine/Manitoba/SD0203/2016	H1α-3
CY246747	Swine	H1N2	A/swine/Manitoba/SD0209/2016	H1α-3
CY246739	Swine	H1N2	A/swine/Manitoba/SD0211/2016	H1α-3
MK462531	Swine	H1N2	A/swine/Manitoba/SD0213/2017	H1α-3
MK462515	Swine	H1N2	A/swine/Manitoba/SD0216/2017	H1α-3
MK462547	Swine	H1N2	A/swine/Manitoba/SD0218/2017	H1α-3
MK462587	Swine	H1N2	A/swine/Manitoba/SD0236/2017	H1α-3
MK462878	Swine	H1N2	A/swine/Manitoba/SD0241/2017	H1α-3
MK462822	Swine	H1N2	A/swine/Manitoba/SD0302/2018	H1α-3
DQ280236	Swine	H1N2	A/swine/Ontario/48235/04	δ-like HA
DQ280227	Swine	H1N2	A/swine/Ontario/52156/03	δ-like HA
DQ280212	Swine	H1N2	A/swine/Ontario/55383/04	δ-like HA
KM489567	Swine	H1N2	A/swine/Ontario/68/2012	npdm
MK328725	Swine	H1N2	A/swine/Ontario/DM_11/2017	H1α-3
MK328741	Swine	H1N2	A/swine/Ontario/DM_21/2017	β
CY246169	Swine	H1N2	A/swine/Saskatchewan/D0410/2016	H1α-3
CY245584	Swine	H1N2	A/swine/Saskatchewan/SD0136/2016	H1α-3
MK462362	Swine	H1N2	A/swine/Saskatchewan/SD0142/2016	H1α-3
CY246161	Swine	H1N2	A/swine/Saskatchewan/SD0155/2016	npdm
CY246219	Swine	H1N2	A/swine/Saskatchewan/SD0163/2016	npdm
CY250816	Swine	H1N2	A/swine/Saskatchewan/SD0204/2016	H1α-3
MK462758	Swine	H1N2	A/swine/Saskatchewan/SD0204/2016	H1α-3
MK462419	Swine	H1N2	A/swine/Saskatchewan/SD0240/2017	H1α-3
MK462918	Swine	H1N2	A/swine/Saskatchewan/SD0291/2018	npdm
EPI1815179	Human	H1N2v	A/Alberta/01/2020	H1α-3a variant
A/sw/AB/SD0308/2018	Swine	H1N2	A/swine/Alberta/SD0308/2018	H1α-3a
A/sw/AB/SD0345/2018	Swine	H1N2	A/swine/Alberta/SD0345/2018	H1α-3a
A/sw/AB/SD0427/2019	Swine	H1N2	A/swine/Alberta/SD0427/2019	H1α-3a

NCBI accession no.	Host	Genotype	Strain	Clade
A/sw/AB/SD0473/2019	Swine	H1N2	A/swine/Alberta/SD0473/2019	H1α-3a
A/sw/AB/SD0529/2020	Swine	H1N2	A/swine/Alberta/SD0529/2020	H1α-3a
A/sw/AB/SD0545/2020	Swine	H1N2	A/swine/Alberta/SD0545/2020	H1α-3a
EPI1056725	Human	H1N2v	A/Ohio/24/2017	H1α-3a variant

*HA, hemagglutinin; NCBI, National Center for Biotechnology Information (http://www.ncbi.nlm.nih.gov); npdm, influenza A(H1N1)pdm09-like virus.
